# ASATrans: Adaptive spatial aggregation transformer for cervical nuclei segmentation on rough edges

**DOI:** 10.1371/journal.pone.0307206

**Published:** 2024-07-12

**Authors:** Hualin Sun, Shengyao Hu

**Affiliations:** 1 ChangZhou Vocational Institute Of Mechatronic Technology, ChangZhou, China; 2 ChangZhou Institute Of Technology, ChangZhou, China; University of Exeter, UNITED KINGDOM OF GREAT BRITAIN AND NORTHERN IRELAND

## Abstract

The main characteristic of cervical cytopathy is reflected in the edge shape of nuclei. Existing computer-aided diagnostic techniques can clearly segment individual nuclei, but cannot clearly segment the rough edges of adherent nucleus. Therefore, we propose an effective method (ASATrans) to accurately segment rough cervical nuclei edges by exploring adaptive spatial aggregation methods. ASATrans creates a Multi-Receptive Embedding Layer that samples patches using diverse-scale kernels. This approach provides cross-scale features to each embedding, preventing semantic corruption that might arise from mapping disparate patches to analogous underlying representations. Furthermore, we design Adaptive Pixel Adjustment Block by introducing a long-range dependency and adaptive spatial aggregation. This is achieved through the stratification of the spatial aggregation process into distinct groups. Each group is given an exclusive sampling volume and modulation scale, fostering a collaborative learning paradigm that combines local features and global dependencies. This collaborative approach to feature extraction achieves adaptability, mitigates interference from unnecessary pixels, and allows for better segmentation of edges in the nucleus. Extensive experiments on two cervical nuclei datasets (HRASPP Dataset, ISBI Dataset), demonstrating that our proposed ASATrans outperforms other state-of-the-art methods by a large margin.

## Introduction

Cell nuclei, as a major factor in assessing cervical cytopathology [1, 2], are crucial due to their significant manifestation in cervical cancer lesions [3]. Traditional cervical cancer pathology diagnosis mainly relies on physicians observing the morphological features of nuclei through a microscope [4, 5]. However, this method suffers from problems such as limited field of view and visual fatigue, which can easily lead to misdiagnosis. Recognition of cell nuclei in real scenarios remains challenging for current computer-aided diagnostic techniques [6, 7].

Existing vision transformers have wider receptive fields [8, 9], can effectively model long-distance relationships, and show excellent performance under large-scale training data and sufficient model parameters [10–12]. However, transformers lack some of the inductive biases inherent in convolutional neural networks (CNNs), and they often require large amounts of data to accurately model relationships, making them generally less performant than CNN models. This is especially true in real-life scenarios, where cervical cells are numerous, distributed in clusters, and have a stacking phenomenon. There are almost no studies that have applied transformer to the field of cervical cell nuclei segmentation. This is because directly applying existing transformer models may lead to poor segmentation accuracy and blurred nuclei edges [11, 13, 14]. The purpose of this paper is to improve the transformer model on small-scale datasets by exploring adaptive spatial aggregation methods to effectively segment rough cervical cancer cell nuclear edges [15, 16].

The primary distinction between vision transformers and Convolutional Neural Networks (CNNs) lies in their approach: vision transformers partition the image into patches and present a sequence of linear embeddings derived from these patches as input to the transformer block. Nevertheless, due to the varying scales of objects in different images [17, 18], the use of fixed-size patches often encounters challenges in capturing comprehensive local structures associated with objects. The rigidity of fixed patches introduces the risk of compromising semantic information, consequently resulting in a decline in performance. Existing segmentation methods pay little attention to this. To address this, We introduce a novel module termed the Multi-Receptive Embedding Layer (MREL), positioned at the initiation of each stage. MREL accepts the output (or input image) from the preceding stage and employs diverse-scale kernels to sample patches. This methodology imparts cross-scale features to each embedding, mitigating the potential semantic corruption arising from the assignment of disparate patches to analogous underlying representations. Consequently, MREL possesses the capability to reconfigure otherwise isolated patches into overlapping patches with varied receptive field sizes. This capability compensates for the loss of image information at the edges of patches due to simplistic patch segmentation and averts semantic corruption resulting from the convergence of different patches into similar latent representations.

Existing multi-attention mechanisms treat all pixels equally, which may lead to optimization bias towards smoothing the inner regions while underestimating the boundary pixels. This can unbalance foreground and background information, resulting in rougher predicted mask boundaries that do not align well with the boundaries of real objects [19–25]. To address this problem, we design Adaptive Pixel Adjustment Block(APAB) by introducing a long-range dependency and adaptive spatial aggregation. This is achieved through the stratification of the spatial aggregation process into distinct groups. Each group is given an exclusive sampling volume and modulation scale, fostering a collaborative learning paradigm that combines local features and global dependencies. This collaborative feature extraction effort enables adaptivity, mitigating interference from unnecessary pixels and allowing for better segmentation of edges in nuclei.

In summary, the contributions of this paper are:

1. Aiming at the problem that existing methods cannot achieve good segmentation results on small-scale data sets, we propose a novel transformer model—ASATrans, which generates finer nuclei edge shapes by exploring adaptive spatial aggregation methods.
2. Specifically, Multi-Receptive Embedding Layer in ASATrans samples patches using diverse-scale kernels. This approach provides cross-scale features to each embedding, preventing semantic corruption that might arise from mapping disparate patches to analogous underlying representations.
3. In addition, we design Adaptive Pixel Adjustment Block by introducing a long-range dependency and adaptive spatial aggregation. This is achieved through the stratification of the spatial aggregation process into distinct groups. Each group is given an exclusive sampling volume and modulation scale, fostering a collaborative learning paradigm that combines local features and global dependencies.
4. Extensive experiments on two cervical nuclei datasets (HRASPP Dataset, ISBI Dataset), demonstrating that ASATrans outperforms other state-of-the-art methods by a large margin.

## Literature review

In the past decade, Deep Convolutional Neural Networks (CNNs) have been extensively employed for medical image segmentation, consistently exhibiting satisfactory performance. The preference for CNN architectures in numerous medical tasks due to their rapidly converge on modest datasets, yielding commendable accuracy and robustness. Building upon the success of transformers developed for Natural Language Processing (NLP) [26–30], researchers have tailored specific vision transformers for visual tasks, leveraging their potent attention mechanisms. Notably, ViT [9] and DeiT [31] successfully adapted the original transformer to vision domains, yielding impressive outcomes. Subsequent innovations, such as PVT [17], Swin [10], and ViTAE [32], introduced the pyramid structure to vision transformers, substantially reducing the number of patches in underlying layers. Furthermore, these advancements extended the applicability of vision transformers to diverse visual tasks, including object detection and segmentation. In addition, these advances have successfully extended Transformer to various other visual tasks [33], including detection, classification, segmentation, etc. Task scenarios include liver tumor segmentation [34], cell segmentation [35], etc.

While vision transformers (ViTs) [36, 37] have demonstrated exceptional performance on large datasets, their efficacy tends to diminish when trained on smaller datasets, possibly attributable to the absence of localized inductive bias in their architecture. Recent investigations [15] have addressed this limitation by introducing locality to the architecture, thereby enabling ViTs to achieve performance comparable to CNNs in scenarios involving smaller datasets. To address the challenge of indistinct edges, Wang et al. [38] proposed the Boundary-Aware Transformer (BAT), incorporating boundary-aware gates in the transformer architecture to leverage prior knowledge about boundaries. BAT was effectively trained with assisted supervision to enhance performance. Additionally, Pu et al. introduced the Transformer-Based Edge Detector (EDTER) [39], employing two distinct phases to extract global context and local cues, which are subsequently fused by a feature fusion module for precise edge prediction. Although these methods exhibit success in diverse domains, the scarcity of foreground pixels in cervical cell nuclei segmentation poses challenges in rapidly establishing a local vision structure. Consequently, there is an urgent need for a transformer model tailored to excel on small-sized cervical cell nuclei segmentation datasets.

## Methodology

**Fig 1:**
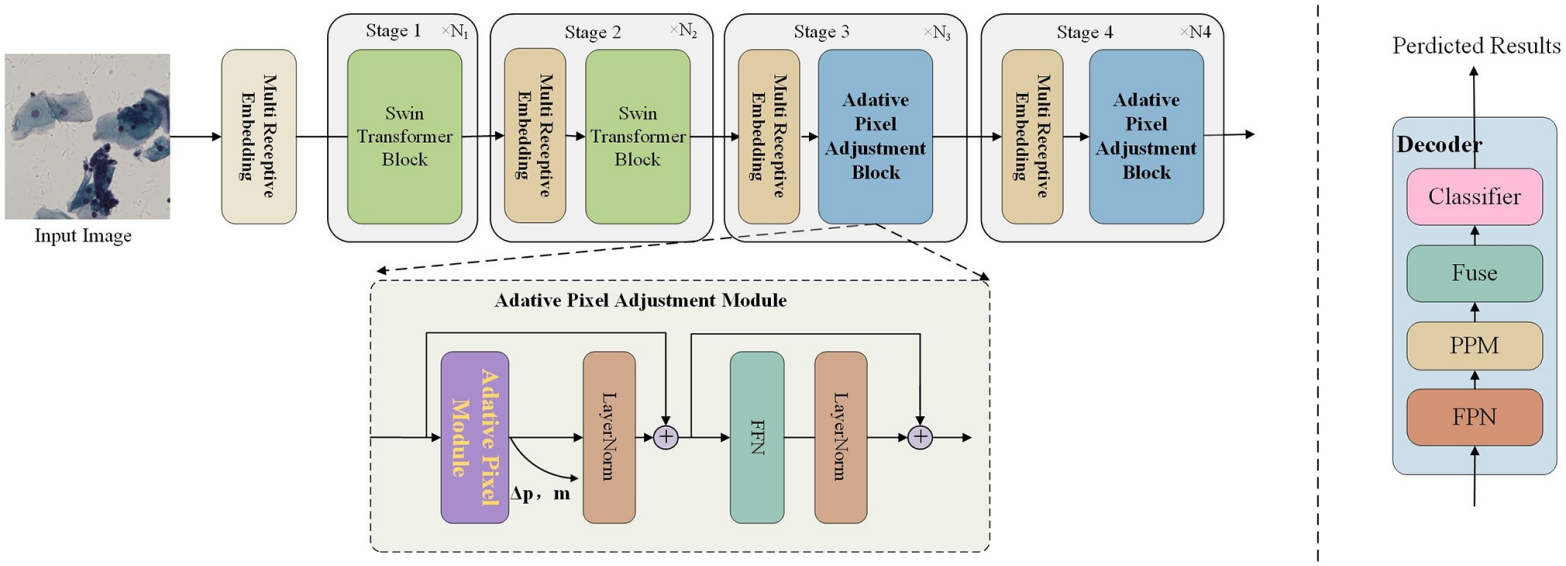
An illustration of the model architecture of ASATrans. The overview of our proposed ASATrans architecture, including the global overview of backbone (above), details of the internal structure of our Adaptive Pixel Adjustment Block (below).

We proposed ASATrans to solve the problem of blurred edge segmentation of transformer on small-scale datasets. The overall structure is shown in Fig 1. The input image first passes through the Multi Receptive Embedding Layer, and then passed through the Swin Transformer Block in stages 1 and 2 and the Adaptive Pixel Adjustment Block and applied in stages 3 and 4. In the decoding part, we use UperNet Head as the decoding head, which mainly includes FPN and PPM modules. Finally, we get the final prediction result through a classifier.

### Adaptive pixel adjustment block

Unlike CNN models, visual transformer has a larger receptive field and excels at modeling long-distance relationships, which shows excellent performance on large datasets. However, since transformer lacks some of the inductive biases that CNNs inherently have, causing it to often require a large amount of data to model relationships, it tends to perform less well than CNN models on undersized medical image datasets. In particular, in the field of cervical cancer cell nuclei segmentation, the small number of datasets and the disparity in the ratio of foreground pixels to background pixels make it difficult for existing transformer models to achieve satisfactory results.

So we designed the Adative Pixel Adjustment Block to replace the traditional transformer block and use it to make up for the shortcomings of convolution and multi-head self-attention. Compared with MHSA whose weights are dynamically adjusted by the input, the Adative Pixel Module is an operator with static weights and strong inductive bias, so that APAB can fully enjoy the advantages of both mechanisms. Due to their highly inductive nature, models composed of regular convolutions may converge faster than VITS and require less training data. The traditional multi-attention mechanism is shown below:

$$\begin{array}{c} Attention\left(Q,K,V\right)=Softmax\left(\frac{QK^{T}}{\sqrt{d_{head}}}V\right) \end{array}$$ (1)

A direct approach to reconcile the disparity between convolutional operations and Multi-Head Self-Attention (MHSA) is to imbue conventional convolution with the capacity for long-range dependencies and adaptive spatial aggregation. Analogous to the DCNv2, this represents a generalized form of traditional convolution. For a given input tensor $w_{k}\in R^{C\times H\times W}$ and the present pixel *p*_0_, the formulation of DCNv2 can be expressed as:

$$\begin{array}{c} y\left(p_{0}\right)=\sum_{p_{n}\in R}W\left(p_{n}\right)\cdot x\left(p_{0}+p_{n}\right) \end{array}$$ (2)

$$\begin{array}{c} y\left(p_{0}\right)=\sum_{p_{n}\in R}W\left(p_{n}\right)\cdot x\left(p_{0}+p_{n}+△p_{n}\right) \end{array}$$ (3)

$$\begin{array}{c} y\left(p_{0}\right)=\sum_{k=1}^{K}w_{k}m_{k}x\left(p_{0}+p_{k}+\Delta p_{k}\right) \end{array}$$ (4)

Here, *K* signifies the overall count of sampling points, with *k* serving as the index for individual sampling points. The notation $w_{k}\in R^{C\times H\times W}$ designates the projection weights associated with the *k*-th sampling point, while $m_{k}\in R$ denotes the modulation scalar corresponding to the *k*-th sampling point. This modulation scalar is subject to normalization through a sigmoid function. Additionally, *p*_*k*_ represents the *k*-th location within the predefined grid sampling, akin to conventional convolutional processes.

$$\begin{array}{c} R=\{(-1,-1),(-1,0),\ldots ,(0,1),(1,1)\} \end{array}$$ (5)

The symbol Δ*p*_*k*_ denotes the displacement corresponding to the k-th grid sampling location. It is discernible from the mathematical expression that, in the context of long-range dependencies, the sampling offset Δ*p*_*k*_ exhibits a degree of flexibility, enabling its interaction with features of both short and long-range characteristics. Moreover, for the purpose of adaptive spatial aggregation, both the sampling offset Δ*p*_*k*_ and the modulation scalar *m*_*k*_ are endowed with learnable attributes and are conditioned by the input variable *x*. It is thereby evident that DCNv2 shares analogous advantageous characteristics with Multi-Head Self-Attention (MHSA), prompting our initiative to construct foundation models of large-scale Convolutional Neural Networks (CNNs) grounded upon this operator.

In order to augment the efficacy of the convolutional structure embedded within the transformer block, we have operationalized the ensuing strategies:

**Weight sharing among convolutional neurons.** In convolution, different convolution neurons have independent linear projection weights, so their parameters and complexity are linearly related to the total number of sampling points. We use the idea of separable convolution to share the weights between neurons, which effectively reduces the auxiliary degree of the model and makes it possible to apply it in large-scale models. We separate the weight *w*_*k*_ in normal convolution into a depth part and a point part. The original position-aware modulation scalar *m*_*k*_ is responsible for the depth part, and the shared projection weight *w* between sample points represents the point part.

**Introducing the multi-group mechanism.** Second, weight sharing just for convolutions is not enough. In MHSA, attention is often divided into multiple groups for calculation. Inspired by this, we also divide the spatial aggregation process of APAB into G groups. The basic idea is similar to the MHSA widely used in transformre, except that each group of ours has a separate sampling offset Δ*p*_*gk*_ and modulation scale *m*_*gk*_. Therefore, different spatial aggregations exist in different groups on a single convolutional layer, so they can better adapt to different downstream tasks and achieve better convergence speed and performance.

**Normalization of the modulation scalar across sampling points.** In order to control the sampling offset in each group not to exceed a reasonable range, we need to normalize the sampling points. Because the gradient in convolution is unstable when training with large-scale parameters or data. The offset of all sample points in APAB may be outside the normal range. In order to solve this problem, we use the improved sigmoid function to normalize the elements of the modulation parameter scalar, which can make the offset parameters more stable during the training process. The original formula is as follows:

$$\begin{array}{c} \text{softmax}(x_{i})=\frac{e^{x_{i}-c}}{\sum_{j=1}^{d}e^{x_{j}-c}}=\frac{e^{x_{i}}e^{-c}}{e^{-c}\sum_{j=1}^{d}e^{x_{j}}} \end{array}$$ (6)

We have made some changes to reduce the computational cost and increase the speed. The specific implementation method is to take the log of the value, as follows:

$$\begin{array}{c} \text{softmax}(x_{i})=\text{log}\frac{e^{x_{i}-c}}{\sum_{j=1}^{d}e^{x_{j}-c}}=x_{i}-c-\text{log}\sum_{j=1}^{d}e^{x_{j}-c} \end{array}$$ (7)

Compared with softmax, using Log-softmax has many advantages, including improved numerical performance and gradient optimization. These advantages are very important for implementation, especially when the computational cost of training the model is high, they can bring very objective benefits. Moreover, the use of log probability has better information theory interpretability.

In order to control the sampling offset in each group not to exceed a reasonable range, we need to normalize the sampling points. Because when training with large-scale parameters or data, the gradient in convolution is unstable. The offset of all sampling points in APAB may exceed the normal range. In order to solve this problem, we use the sigmoid function to normalize the elements of the modulated parameter scalars, so that the sum of all scalars is 1, which can make the offset parameters during the training process more stable.

Combined with the above modifications, the extended DCNv2, can be formulated as:

$$\begin{array}{c} y\left(p_{0}\right)=\sum_{g=1}^{G}\sum_{k=1}^{K}w_{g}m_{gk}x_{g}\left(p_{0}+p_{k}+\Delta p_{gk}\right) \end{array}$$ (8)

Let *G* denote the total number of aggregated groups. In the context of the *g*-th group, $w_{g}\in R^{C\times C^{\prime }}$ represents the position-independent projection weight, where *C*′ = *C*/*G* signifies the dimension of the group. The term $m_{gk}\in R$ pertains to the offset associated with the *k*-th sampling point in the *g*-th group, which is normalized along the *k*-th dimension through the application of the softmax function. The variable $x_{g}\in R^{C^{\prime }\times H\times W}$ denotes the sliced input feature map within the *g*-th group. Furthermore, Δ*p*_*gk*_ represents the offset corresponding to the grid sampling position *p*_*k*_ within the *g*-th group.

The predefined scalar *γ* used to modulate the offset amplitude is empirically set to 0.1, which we believe is unreasonable empirically. Because this limits the offset distance, if a large range of edge distortion is encountered, the degree of deformation will not be enough to cope with large changes. And if you encounter smaller deformations, it will be difficult to identify the degree of distortion. We set *γ* as a variable variable, and its formula is

$$\begin{array}{c} △p_{ij}=\gamma △\hat{p_{ij}}⊙\left(w,h\right) \end{array}$$ (9)

where (w, h) are the width and height of the ROI, by element-wise product with the width and height of the ROI. *γ* is limited between 0.01–0.5, with an initial value of 0.1, which is obtained through adaptive learning. The parameters are continuously adjusted during the training process through the back propagation algorithm to improve the accuracy and generalization ability of the model, and then transform the deformed The amplitude is controlled within a reasonable range, thereby better segmenting the edges of distorted cervical cell nuclei and helping doctors better judge the extent of cancer lesions.

In summary, the APAM operator serves to rectify the limitations of regular convolution with respect to long-distance dependencies and adaptive spatial aggregation.

### Multi receptive embedding layer

The main difference between visual transformers and CNN models is how images are processed. In visual transformers, images are divided into blocks, and these blocks are linearly embedded and passed through transformer blocks. However, this simple patch-based segmentation approach has two issues: (1) Loss of local structures: Regular patches (e.g., 16x16) struggle to capture complete local object structures as object scales vary in different images. (2) Semantic inconsistency: Objects in different images may have different geometric variations (scaling, rotation, etc.), and fixed patch segmentation may capture inconsistent object information, potentially degrading semantics and performance. As a result, some intrinsic inductive bias is lost during image segmentation, leading to inferior performance on small-scale datasets compared to CNN models.

**Fig 2:**
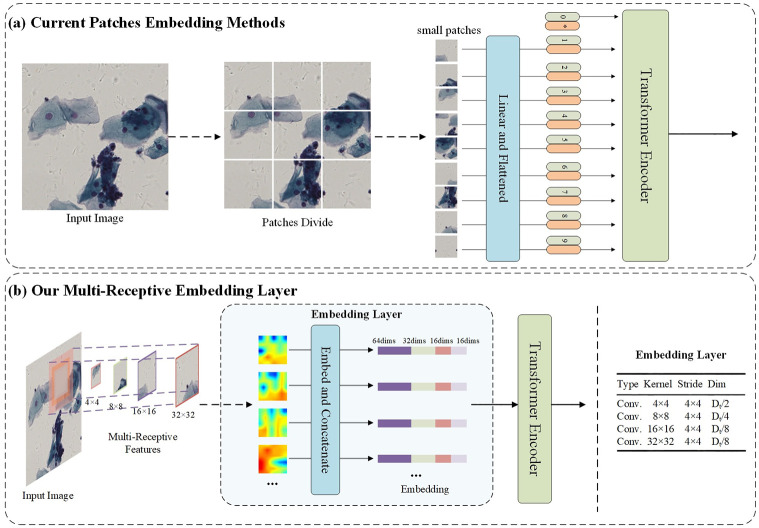
An illustration of consecutive pixel patch moudle. (a) shows current patches embedding methods. (b) shows our multi-receptive embedding layer.

In Fig 2(a), the current patch embedding method divides the image into small patches, linearizes them, and then flattens them before inputting them to the encoder. In contrast, Fig 2(b) shows our approach, which embeds and concatenates multi-sized image patches into a linear representation.

Multi-Resolution Embedded Layers (MRELs) are employed for the generation of input embeddings at each stage of the process. The initial MREL, depicted in Fig 2 and positioned prior to the first stage, accepts the image as its input. Subsequently, it samples patches using four kernels characterized by varying sizes. The step size of these kernels is appropriately adjusted to ensure uniform embedding counts. Notably, these four patches correspond to identical central regions but vary in scales. Ultimately, these patches undergo projection and consolidation into a unified embedding, a process typically executed through the utilization of four convolutional layers.

Dealing with cross-scale embeddings poses the challenge of selecting the right projection dimension for each scale. The computational cost of a convolutional layer scales with *K*^2^ × *D*^2^, where K is the kernel size, and D represents the input/output dimensions when they are equal. This means that larger kernels consume more computational resources compared to smaller ones for the same dimension. To efficiently manage the computational budget of the Multi-Resolution Embedding Layer (MREL), we assign lower dimensions to larger kernels and higher dimensions to smaller kernels. Specific allocation rules, along with a 128-dimensional example, are provided in sub-tables within Fig 2. Our approach significantly reduces computational overhead without significantly affecting the model’s performance, compared to the conventional practice of evenly distributing dimensions. Similar processes are followed in the cross-scale embedding layers in other stages. As shown in Fig 1, MRELs in stages 2/3/4 utilize two different kernel sizes (2x2 and 4x4). Additionally, in stages 2/3/4, the MREL span is set to 2x2 to create a pyramid structure, effectively reducing the number of embeddings to one-fourth.

## Experiments

### Dataset and metric

**Fig 3:**
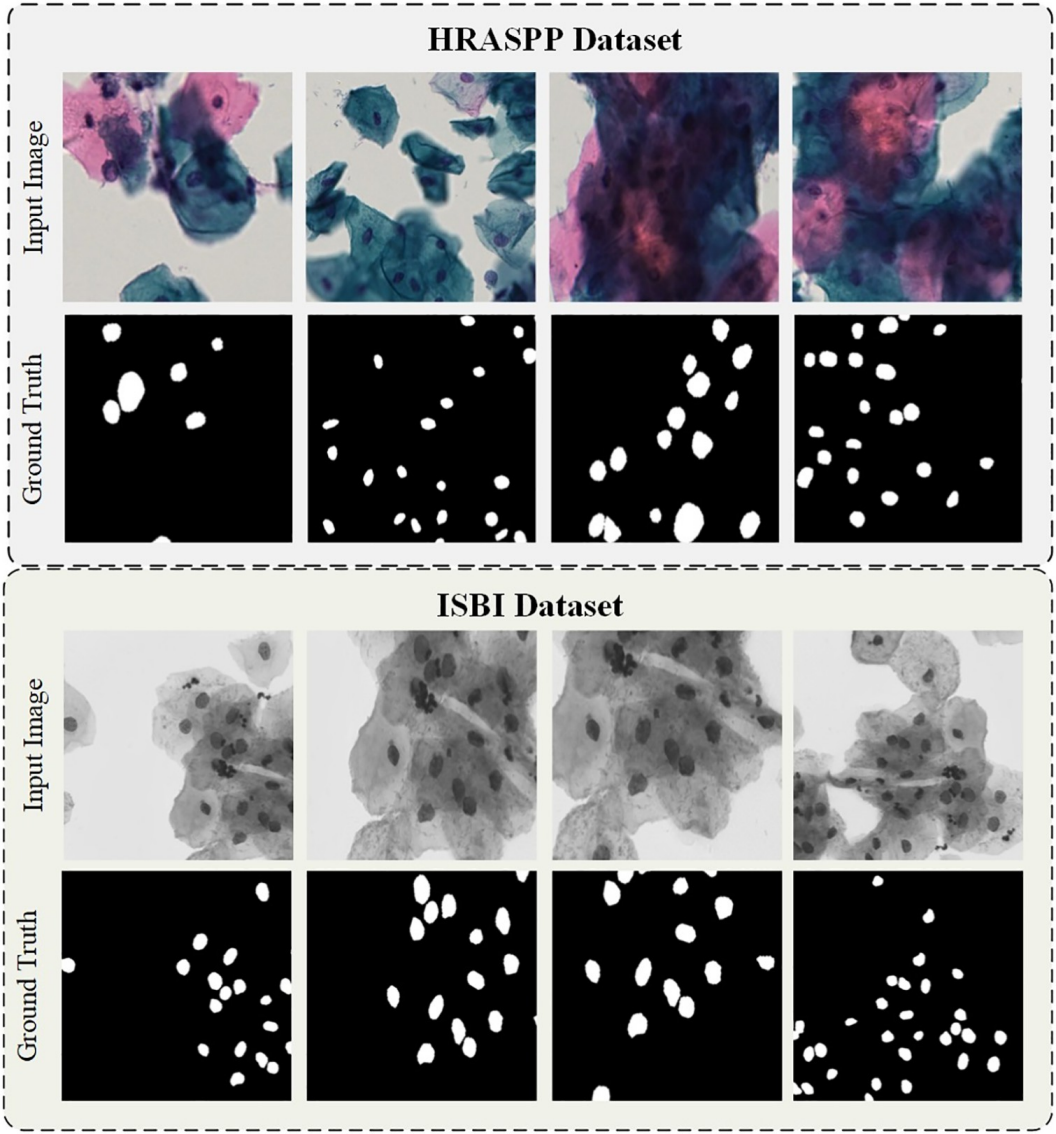
Display of dataset image.

This article presents experimental investigations conducted on both HRASPP datasets [3] and publicly available ISBI datasets [22]. Each dataset exhibits distinctive characteristics. The internally generated dataset is characterized by an abundance of cell clusters or stacks, posing challenges in the segmentation process. Notably, a mere 0.01% of pixels within each image correspond to nuclei, as shown in Fig 3. In contrast, the ISBI dataset involves cervical cells obtained through a Pap smear by a skilled medical professional, with subsequent presentation of slide images under a microscope. While the ISBI dataset also exhibits cell adhesion, it is observed to be of a milder degree. Furthermore, there is no substantial size difference in cell nuclei when compared to our proprietary dataset.

In the evaluation of the performance of our ASATrans model, three commonly utilized metrics were chosen: Intersection over Union (IoU), Dice coefficient, and Pixel Accuracy (PA). IoU and Dice coefficient are frequently employed in the assessment of medical image segmentation, as they provide direct quantification of pixel overlap between predicted outcomes and ground truth labels. Furthermore, Pixel Accuracy (PA) denotes the proportion of accurately classified pixels relative to the total pixel count.

These metrics are formally defined as follows:

$$\begin{array}{c} IoU=\frac{TP}{TP+FP+FN} \end{array}$$ (10)

$$\begin{array}{c} Dice=\frac{2TP}{2TP+FP+FN} \end{array}$$ (11)

$$\begin{array}{c} PA=\frac{TP+TN}{TP+TN+FT+FN} \end{array}$$ (12)

### Training details

The experiments were implemented using the PyTorch framework and executed on an NVIDIA GeForce RTX 3080. All methodologies underwent training with a Batch Size of 2 and employed SGD as the optimizer. The initial learning rate was established at 0.001, with a momentum of 0.9 and a weight decay of 0.0005.

The training process incorporated **Multiple Losses**, specifically CrossEntropyLoss and DiceLoss, applied to the datasets. The ratio was set at 3. Data augmentation techniques, including flipping, rotating, and cropping operations, were applied to augment the three datasets. The input model operated on images with dimensions of 512 × 512, while the crop size for the transformer model was set to 224 × 224. Pre-training utilized the ImageNet-1k dataset, and model performance was evaluated on the official test set provided by the dataset itself. A total of 360,000 iterations were conducted during the training phase.

### Comparison with other methods

**Fig 4:**
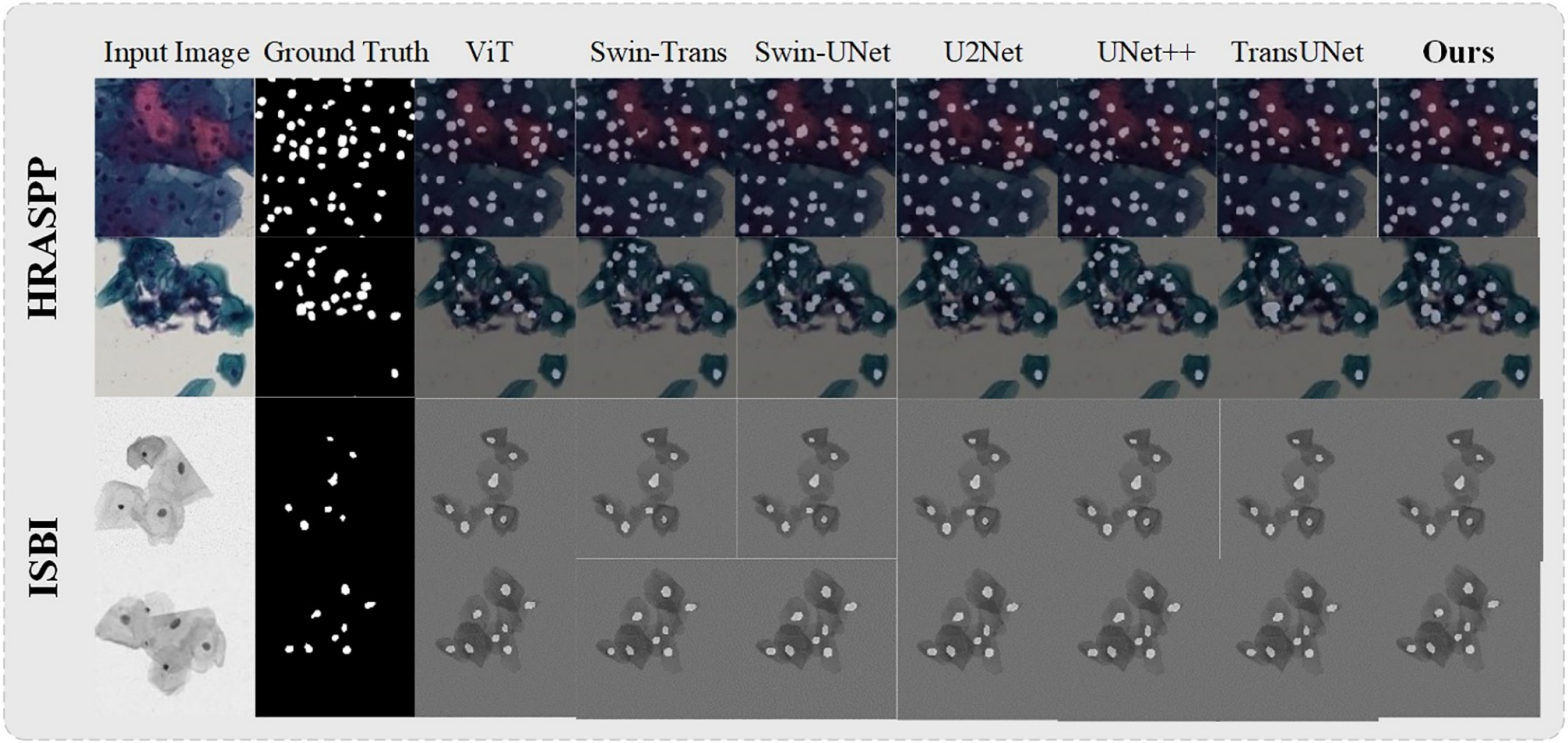
Display of transformer-based methods prediction results. Each dataset presents two nuclei images and ground truth images.

**Fig 5:**
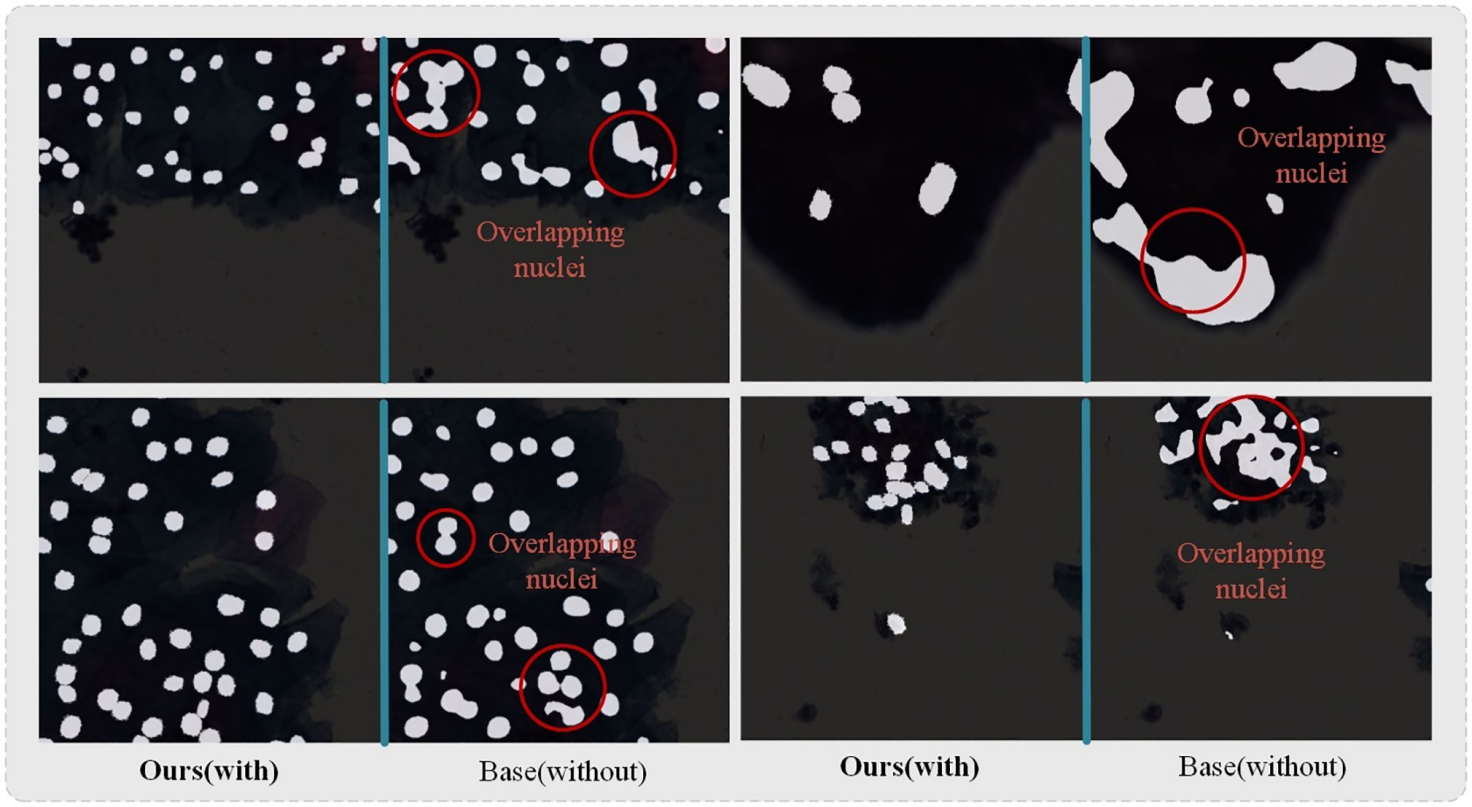
Refinement effect of ASATrans on edge. Left is the segmentation result with ASATrans, right is the segmentation result without ASATrans.

To show the efficacy of ASATrans, a comparative analysis was conducted against several state-of-the-art methods using two distinct datasets. The selected methods encompassed Vision Transformer [9], Swin Transformer [10], Swin Unet [40], U2NET [41], UNET++ [42], and TransUNET [43]. To ensure equitable comparisons, all models were executed within the same configuration environment. The outcomes of these comparisons are presented in Tables 1 and 2, while visual contrasts are depicted in Figs 4 and 5.

**Table 1:** Quantitative comparison of different excellent methods on HRASPP dataset.

Methods	Input Size	Crop Size	Params(M)	IoU	Dice	PA
ViT	512^2^	224^2^	142	0.4077	0.5792	0.5449
Swin-Trans	512^2^	224^2^	120	0.4629	0.6329	0.5709
Swin-UNet	512^2^	224^2^	79	0.4552	0.6256	0.5335
NucleiSegNet	512^2^	256^2^	93.54	0.4943	0.6866	0.5735
U2Net	512^2^	256^2^	44.63	0.5034	0.6697	0.5771
UNet++	512^2^	256^2^	35	0.4761	0.6451	0.5412
OCR	512^2^	256^2^	56.75	0.5058	0.6718	0.6124
HR-AS	512^2^	256^2^	71.23	0.5064	0.6723	0.5923
TransUNet	512^2^	224^2^	86	0.4958	0.6629	0.5854
**Ours**	512^2^	224^2^	63.71	**0.5123**	**0.6732**	**0.5991**

**Table 2:** Quantitative comparison of different excellent methods on ISBI Dataset.

Methods	Input Size	Crop Size	Params(M)	IoU	Dice	PA
ViT	512^2^	224^2^	142	0.7698	0.8699	0.9627
Swin-Trans	512^2^	224^2^	120	0.8628	0.9264	0.9484
Swin-UNet	512^2^	224^2^	79	0.8216	0.9021	0.9532
NucleiSegNet	512^2^	256^2^	93.54	0.8456	0.9261	0.9475
U2Net	512^2^	256^2^	44.63	0.8321	0.9082	0.9480
UNet++	512^2^	256^2^	35	0.8474	0.9174	0.9493
OCR	512^2^	256^2^	56.75	0.8457	0.9163	0.9541
HR-AS	512^2^	256^2^	71.23	0.8498	0.9188	0.9776
TransUNet	512^2^	224^2^	86	0.8581	0.9236	0.9569
**Ours**	512^2^	224^2^	63.71	**0.8779**	**0.9362**	**0.9775**

Across two datasets with disparate characteristics (HRASPP dataset and the ISBI dataset), the results in Tables 1 and 2 consistently indicate the superior performance of our proposed method when compared to both CNN-based and Transformer-based models. This observation underscores the notable potential of our model for the segmentation of cervical cell nuclei edges, particularly in the context of limited dataset sizes.

Specifically, on HRASPP dataset, cervical cell nuclei often appear in cell clusters, leading to cell stacking, overlapping, and difficult segmentation. The CNN-based U2Net and Unet++ models converge quickly and fluctuate stably, and can quickly learn the morphological features of the cell nuclei, achieving notable results, with the best model achieving an IoU of 0.5034. In contrast, the Transformer-based model converges slower and fluctuates more, with a longer training cycle, and due to the lack of the CNN-based model’s inherent of inductive biaes, their performance is often inferior to that of CNN-based models. For example, the ViT model only achieved an IoU of 0.4077, the worst performance on our dataset. However, our ASATrans model provides finer segmentation of cell nucleus edges with comparable model sizes by dynamic adaptive spatial aggregation, which allows the input patch embedding to contain more local detail information, and APAM to bias the transformer’s attention more towards the foreground. Ultimately, our model achieves 0.89% higher IoU performance and 0.35% higher Dice performance than the next best CNN-based model, and 1.65% higher IoU performance and 1.03% higher Dice performance than the next best Transformer-based model. This also proves the effectiveness of our module design.

On the ISBI dataset, all models perform well with very clear nuclei and good contrast, despite the presence of cell stacking. In this case, the model structure based on Transformer can better establish long-distance dependency and fully utilize the performance of Transformer. As can be seen from Table 2, compared with the next best model, the model in this paper achieves 1.51% and 0.98% improvement in IoU and Dice metrics, respectively.

Combining the results of the two individual datasets, we find that the CNN-based model usually converges faster and works better than the Transformer-based model on HRASPP dataset, which has severe nucleus stacking, cell aggregation, and large picture differences. And on ISBI and Herlev, two small datasets with little difference, Transformer can fully utilize the performance. Our designed A and B modules perform adequately on all three datasets to better model cell nucleus images. ASATrans can improve the accuracy of spatial localization of small-size targets such as cell nuclei, act as a refinement of segmentation edges, and enhance the robustness of the model. These optimizations have a significant role in improving the performance in the task of cell nucleus image segmentation.

### Ablation studies

Taking the ISBI dataset as an example, we have conducted experimental discussions on the selection of hyperparameters and ablation experiments under various settings to validate the effectiveness of the proposed individual modules for segmentation.

In order to demonstrate the effectiveness of the proposed modules, we conducted comparison experiments on the ISBI dataset using various combinations as shown in Table 3. Based on the results reported in Table 3, it is easy to see that the components in ASATrans are compatible with each other, while each component contributes to the improvement of segmentation rate.

**Table 3:** Quantitative comparison of different excellent methods on ISBI dataset.

Components		Results		
APAB	MREL	IoU	Dice	PA
x	x	0.8628	0.9264	0.9484
√	x	0.8698	0.9302	0.9507
x	√	0.8718	0.9334	0.9594
√	√	**0.8779**	**0.9362**	**0.9775**

Specifically, we compare the segmentation performance of the base model in the first row and the second row with the addition of the APAB module. Observe that the addition of APAB module improves IoU, Dice and PA by 0.7%, 0.38% and 0.23%, respectively. This indicates that the new embedding can maintain the local continuity of the pixels around the patch, and will not roughly break up the complete image into split chunks as in the case of plain block segmentation, thus avoiding the loss of image information at the edges of the block and maintaining the intrinsic scale invariance of the image. Compared to adding module APAB, comparing the segmentation performance of the base model in the first row and the third row, we find that the performance improvement is more with the addition of module MREL. The IoU and Dice are improved by 0.9% and 0.7%, respectively. This is that the APAM operator compensates for the shortcomings of regular convolution in terms of long distance dependence and adaptive spatial aggregation; Compared with common attention-based operators such as MHSA and closely related deformable attention, this operator inherits the inductive bias of convolution, which makes our model more efficient, with less training data and shorter training time; this operator is based on sparse sampling, which is more efficient than previous methods such as MHSA and heavily parameterized methods such as the large kernel with parameterization is more computationally and memory efficient. MREL supplements the image information lost at patch edges due to plain patch partitioning and prevents the semantic corruption caused by mapping different patches to similar latent representations. Comparing lines 2, 3, and 4, adding two modules results in greater performance improvement compared to adding one module. This demonstrates that the components in ASATrans are compatible with each other and that each component contributes to improving segmentation performance. Furthermore, it shows that our proposed ASATrans effectively enhances the segmentation performance of cervical cancer cell nucleus edges on small datasets.

### Hyperparameter discussion

Multi-group (head) design first appeared in group convolution, which is widely used in MHSA of transformers and used with adaptive spatial aggregation to effectively learn richer information from different representation subspaces at different locations. Inspired by this, we divide the spatial aggregation process into G groups, each group has separate sampling offsets and modulation scales, so different groups on a single convolutional layer can have different spatial aggregation patterns, thus providing better performance for downstream tasks. Strong functionality.

In order to determine the optimal hyperparameter G, we set it to groups 2, 3, 4, 6, and 12 respectively. The experimental results are shown in Table 4.

**Table 4:** Hyperparameter discussion.

Group Number	Improved(%)
2	0.13
3	0.24
**4**	**0.33**
6	0.323
12	0.322

Experimental results show that when G is set to 2, the performance improvement is minimal, only 0.12. However, setting the number of spatial aggregation groups to 4 results in the best performance improvement, up to 0.33. Therefore, we conclude that 4 is the optimal number of groups. This choice is similar to the spatial pyramid structure, where people usually aggregate 4 feature maps of different sizes to obtain comprehensive information. The reason we didn’t choose 5 is that it is difficult to divide. Additionally, when the number of sets increases to 6 or 12, performance drops to about 0.32. We believe this is because aggregating a larger number of groups leads to information redundancy, thereby reducing performance. In addition, choosing a larger number of groups will increase the computational complexity and may lead to the problem of exploding gradients. Therefore, choice 4 considers both speed and accuracy.

### Visualization

#### Segmentation results

Fig 4 shows the visualization results of the segmentation prediction, with two images selected for visualization for each dataset. Observing the visualization results of rows 2, 3, and 4 in Table 4, it can be seen that the visualization results of Transformer models with poorer performance (e.g., Vision Transformer) are also very rough, and the visualization results reflect the performance performance of the models very well. In contrast, as seen in the last column, our model has the best visualization results, with higher segmentation accuracy and finer segmentation of edges.

To further illustrate the improved effect of our proposed APAB on edge segmentation. We compare our model with the benchmark model and show the visualization results. It can be clearly observed that the originally adherent nuclei edges become clearer in the left-right comparison plots in Fig 5. The edges of Overlapping nuclei tend to be adherent and unclear, and we can observe in the region marked by the red box in Fig 5 that the overlapping boundaries segmented by ASATrans are more clearer than the edges segmented by the benchmark model as the training progresses.

#### Convergence and stability

**Fig 6:**
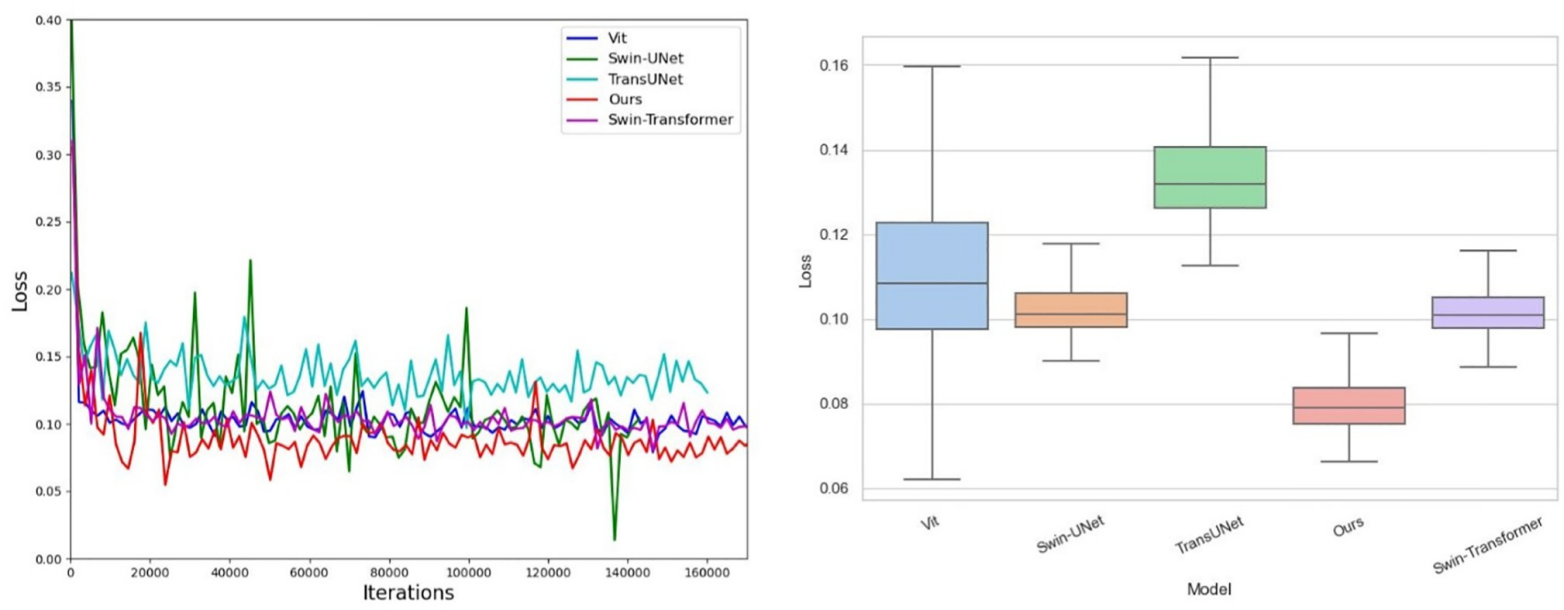
Effect on the convergence and stability.

The ASATrans we proposed can effectively improve the stability of the model and accelerate the convergence of the model. The proposed MREL module avoids the loss of local structure information through multi-scale feature extraction and accelerates the convergence of the model. At the same time, the inherent inductive bias is supplemented to enhance semantic consistency and make model training more stable. As shown in Fig 6, red represents our model. It is obvious that our model is more stable than other models.

#### Attention map

**Fig 7:**
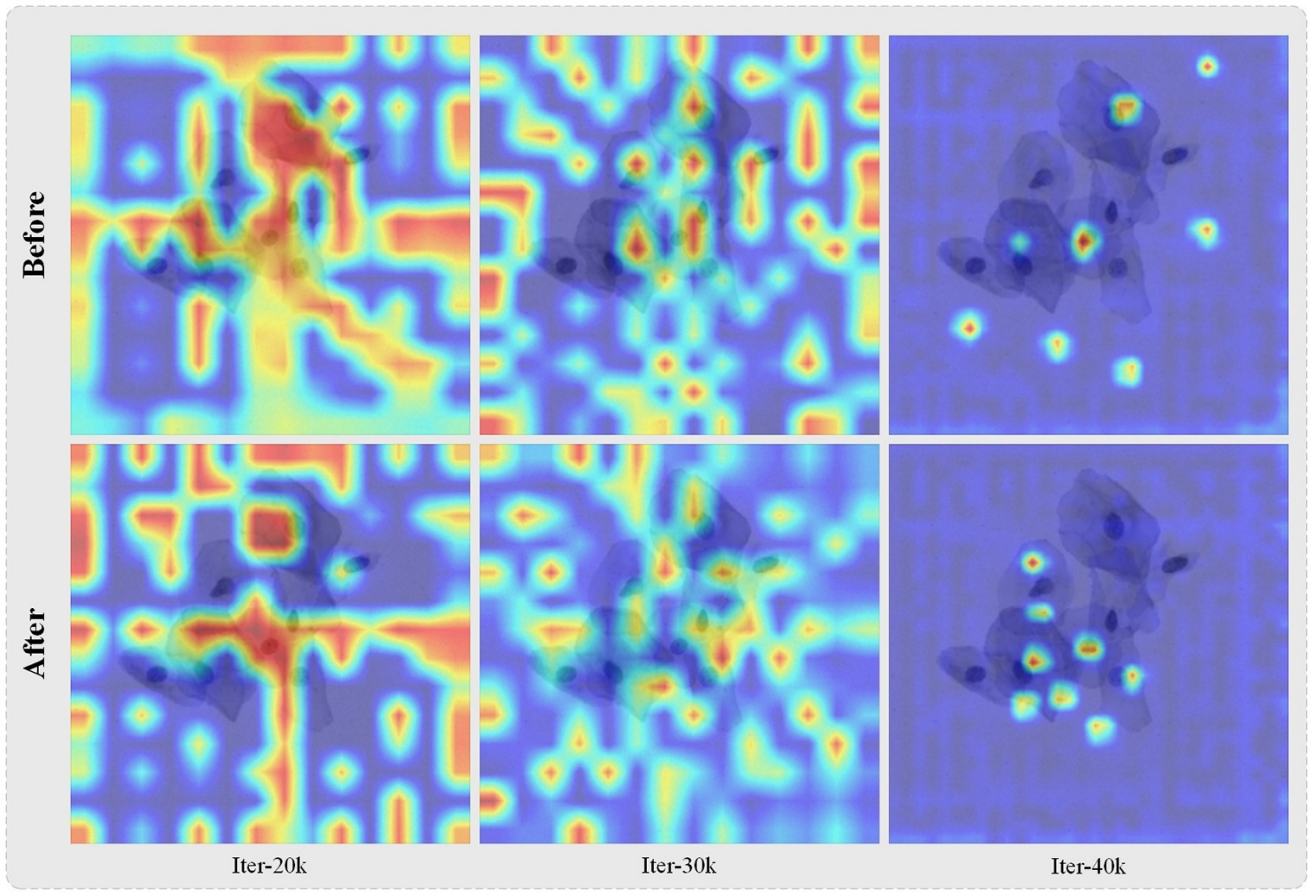
Effect of APAB on attention map. Above is the attention map without APAB, below is the attention map with APAB.

To further illustrate that the APAB does indeed bias attention that would otherwise be focused on the background more towards the foreground, we used GradCAM to visualize the model’s attention. We visualized the 2D activations by weighting the 2D activations by the average gradient and selecting the maximum value channel. The first row of Fig 7 shows the distribution of attention before the addition of the APAB module, and it can be seen that the attention is scattered and much of it is focused on the background. Whereas after the addition of the APAB module, as shown in the second row of Fig 7 the attention is shifted from the background to the foreground, focusing more on the region of the nucleus clusters. Comparing columns 1, 2, and 3 in the figure, we can observe that as the training progresses, Transformer’s attention becomes more refined, better segmenting the edges of the cell nuclei. This further proves the effectiveness of APAB.

## Conclusions

In this paper, we delve into how the transformer model can be improved to finely segment blurred cell nuclei edges on small-scale datasets. First, we observe that the existing transformer model loses edge information when crudely dividing an image into small patches, making it difficult to quickly establish long-distance dependencies, which is detrimental to model convergence and edge segmentation. In addition, when extracting image features, the transformer’s attention tends to focus too much on the background and ignores the important foreground information. To address these issues, we propose a simple yet effective transformer framework named ASATrans, which learns sparse attention in a data-dependent manner and models geometric transformations to bias the attention more from the background to the foreground. ASATrans effectively improves the accuracy of edge segmentation of cell nuclei. We applied ASATrans to the difficult task of cell nuclei segmentation with small datasets and obtained finer cell nuclei segmentation edges. Numerous experiments demonstrate the effectiveness of our ASATrans model, which performs better and achieves significant improvement compared to other baseline models.

## Supporting information

S1 Fig Original pathological images of Figs 1–5 and 7 in the main body. (ZIP)
