# Supplementary figures and images for: ASATrans: Adaptive spatial aggregation transformer for cervical nuclei segmentation on rough edges
Source: PLoS One. 2024 Jul 12;19(7):e0307206. doi: 10.1371/journal.pone.0307206 (PMC11244805; doi:10.1371/journal.pone.0307206)

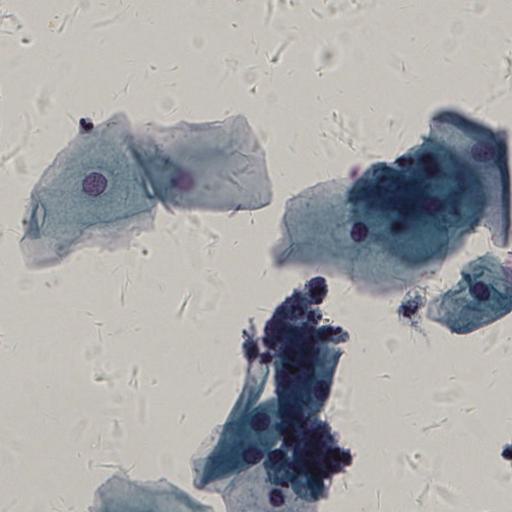

Supplement: S1 Fig — (ZIP) [file pone.0307206.s001.zip › S1.Fig/Fig1.jpg]

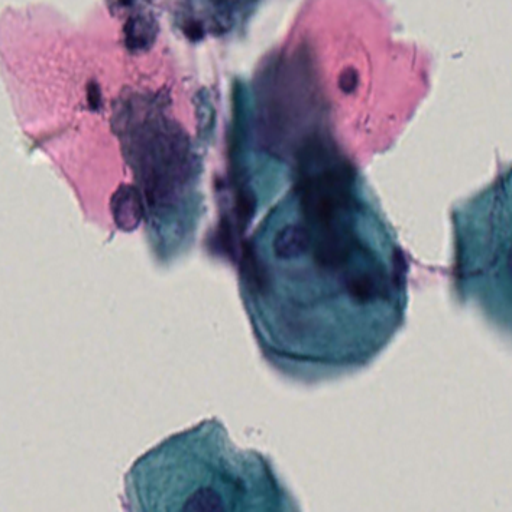

Supplement: S1 Fig — (ZIP) [file pone.0307206.s001.zip › S1.Fig/Fig3.1.png]

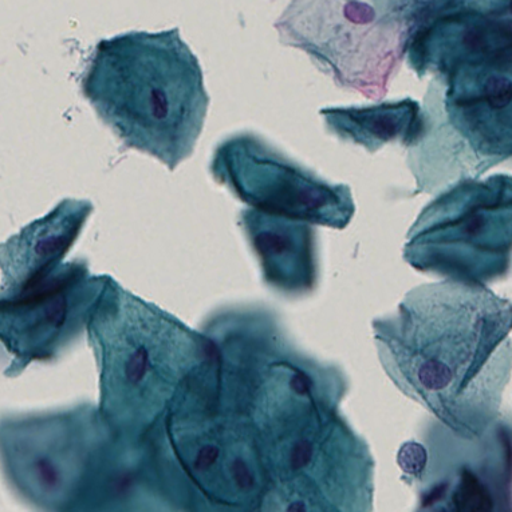

Supplement: S1 Fig — (ZIP) [file pone.0307206.s001.zip › S1.Fig/Fig3.2.png]

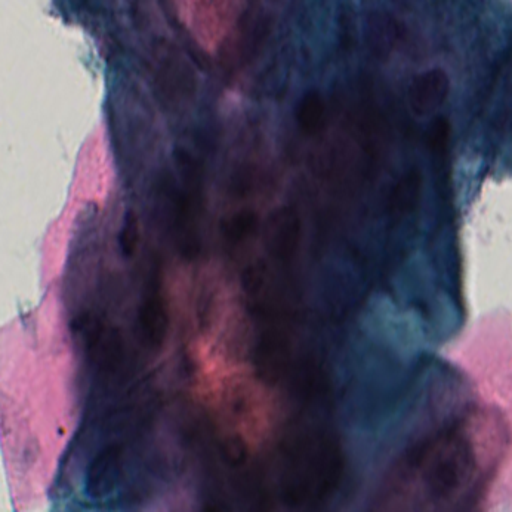

Supplement: S1 Fig — (ZIP) [file pone.0307206.s001.zip › S1.Fig/Fig3.3.png]

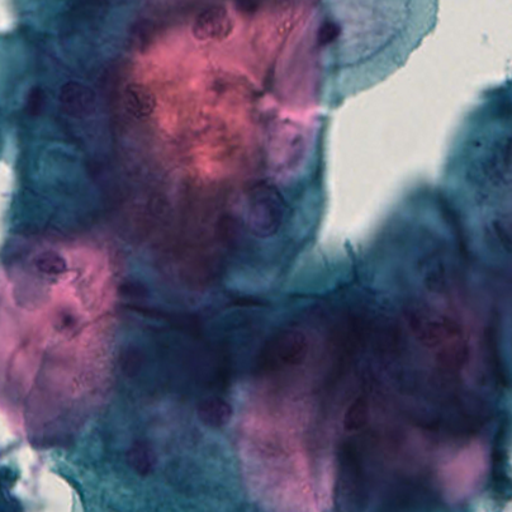

Supplement: S1 Fig — (ZIP) [file pone.0307206.s001.zip › S1.Fig/Fig3.4.png]

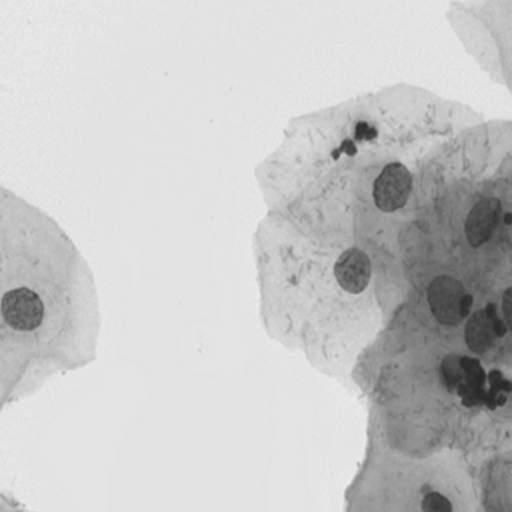

Supplement: S1 Fig — (ZIP) [file pone.0307206.s001.zip › S1.Fig/Fig3.5.png]

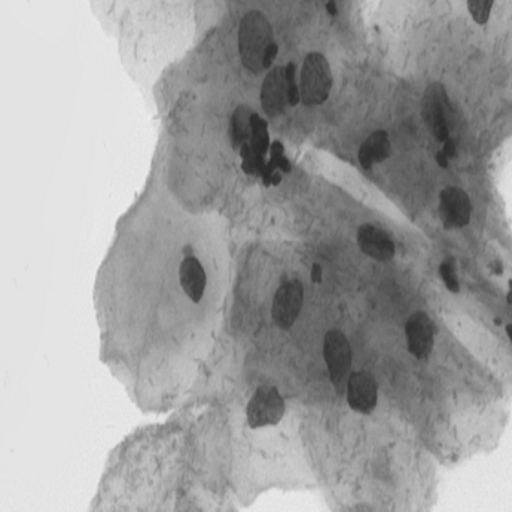

Supplement: S1 Fig — (ZIP) [file pone.0307206.s001.zip › S1.Fig/Fig3.6.png]

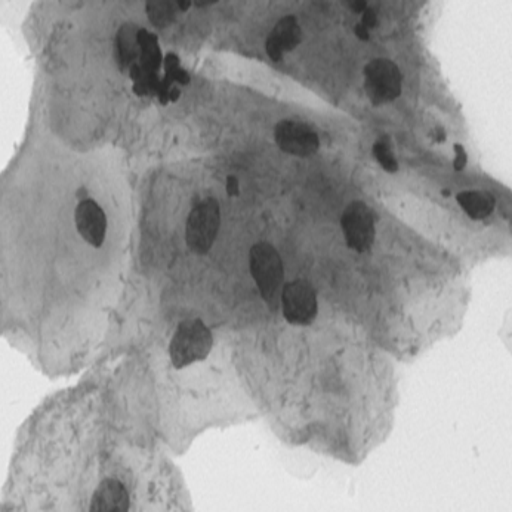

Supplement: S1 Fig — (ZIP) [file pone.0307206.s001.zip › S1.Fig/Fig3.7.png]

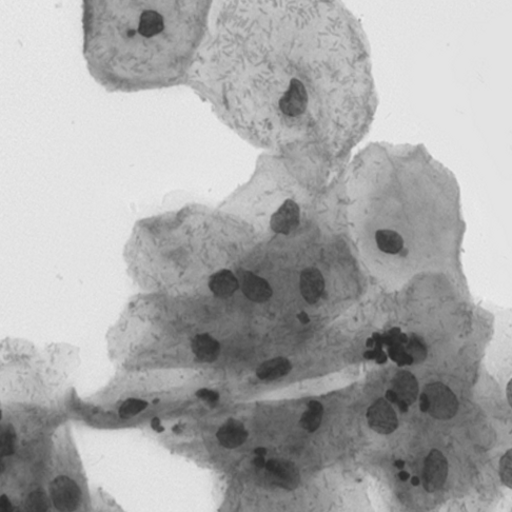

Supplement: S1 Fig — (ZIP) [file pone.0307206.s001.zip › S1.Fig/Fig3.8.png]

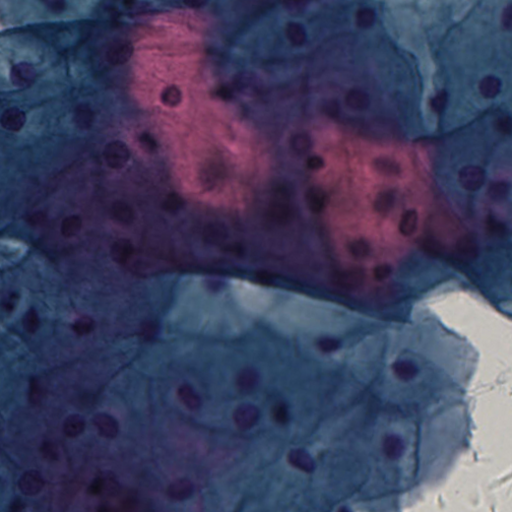

Supplement: S1 Fig — (ZIP) [file pone.0307206.s001.zip › S1.Fig/Fig4.1.png]

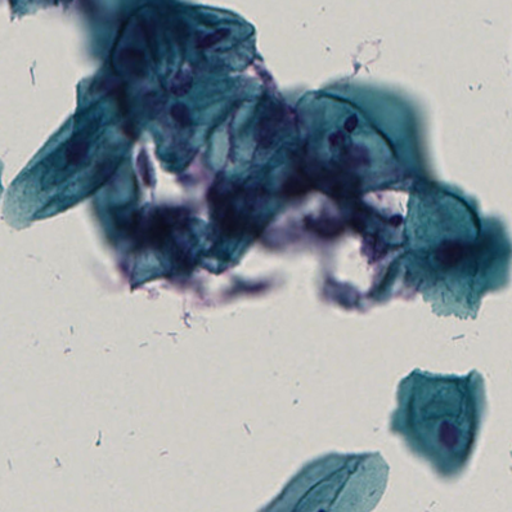

Supplement: S1 Fig — (ZIP) [file pone.0307206.s001.zip › S1.Fig/Fig4.2.png]

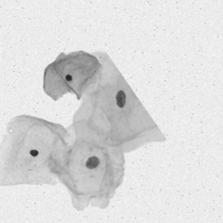

Supplement: S1 Fig — (ZIP) [file pone.0307206.s001.zip › S1.Fig/Fig4.3.png]

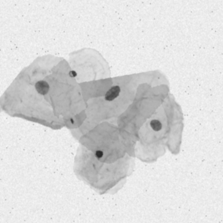

Supplement: S1 Fig — (ZIP) [file pone.0307206.s001.zip › S1.Fig/Fig4.4.png]

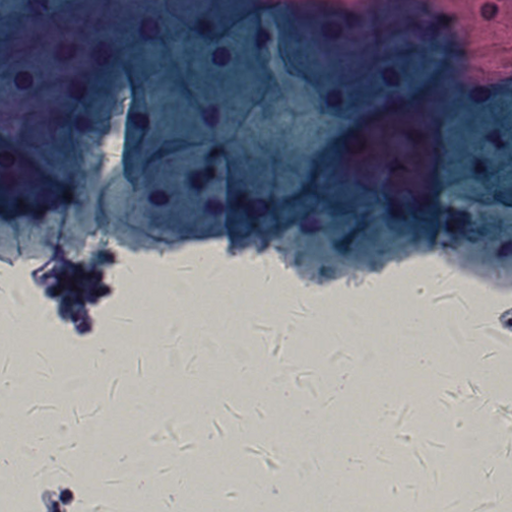

Supplement: S1 Fig — (ZIP) [file pone.0307206.s001.zip › S1.Fig/Fig5.1.png]

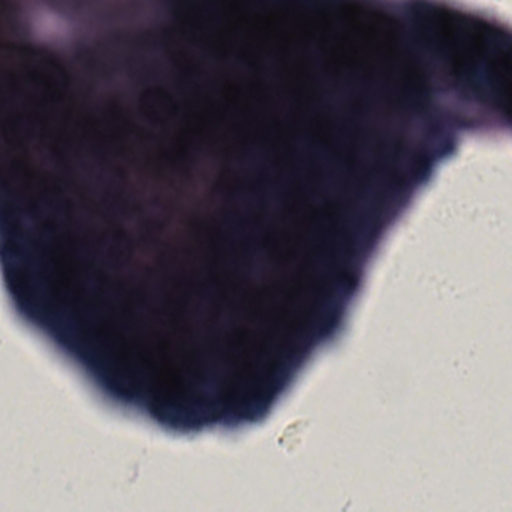

Supplement: S1 Fig — (ZIP) [file pone.0307206.s001.zip › S1.Fig/Fig5.2.png]

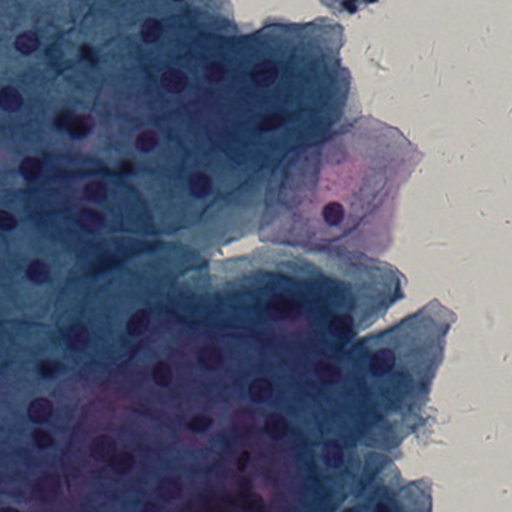

Supplement: S1 Fig — (ZIP) [file pone.0307206.s001.zip › S1.Fig/Fig5.3.png]

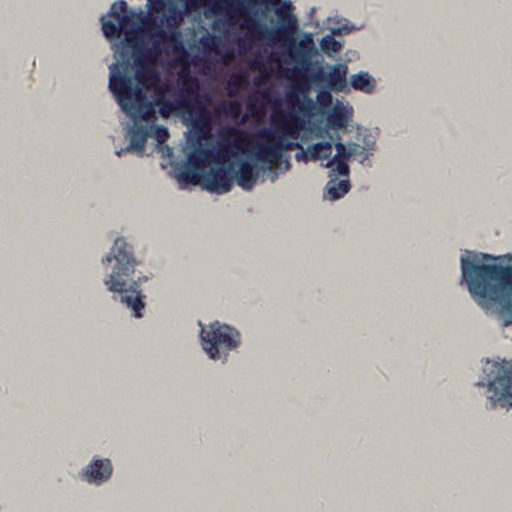

Supplement: S1 Fig — (ZIP) [file pone.0307206.s001.zip › S1.Fig/Fig5.4.png]

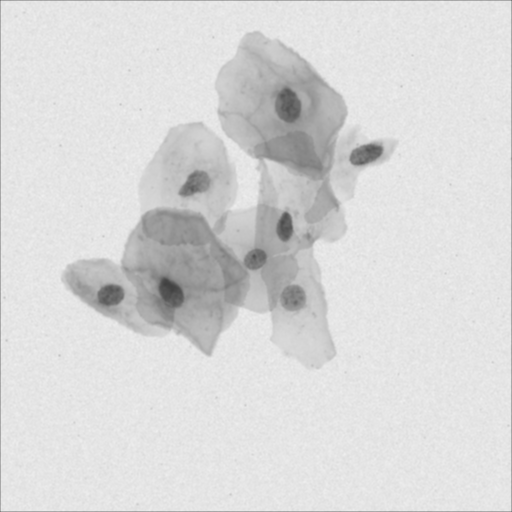

Supplement: S1 Fig — (ZIP) [file pone.0307206.s001.zip › S1.Fig/Fig7.png]
